# *Fusobacterium* Genomics Using MinION and Illumina Sequencing Enables Genome Completion and Correction

**DOI:** 10.1128/mSphere.00269-18

**Published:** 2018-07-05

**Authors:** S. Michelle Todd, Robert E. Settlage, Kevin K. Lahmers, Daniel J. Slade

**Affiliations:** a Department of Biomedical Sciences and Pathology, Virginia-Maryland College of Veterinary Medicine, Blacksburg, Virginia, USA; b Advanced Research Computing, Virginia Polytechnic Institute and State University, Blacksburg, Virginia, USA; c Department of Biochemistry, Virginia Polytechnic Institute and State University, Blacksburg, Virginia, USA; University of Kentucky

**Keywords:** cancer, colorectal cancer, *Fusobacterium*, *Fusobacterium nucleatum*, Illumina, MinION

## Abstract

Understanding the virulence mechanisms of human pathogens from the genus *Fusobacterium* has been hindered by a lack of properly assembled and annotated genomes. Here we report the first complete genomes for seven *Fusobacterium* strains, as well as resequencing of the reference strain Fusobacterium nucleatum subsp. nucleatum ATCC 25586 (total of seven species; total of eight genomes). A highly efficient and cost-effective sequencing pipeline was achieved using sample multiplexing for short-read Illumina (150 bp) and long-read Oxford Nanopore MinION (>80 kbp) platforms, coupled with genome assembly using the open-source software Unicycler. Compared to currently available draft assemblies (previously 24 to 67 contigs), these genomes are highly accurate and consist of only one complete chromosome. We present the complete genome sequence of F. nucleatum subsp. nucleatum ATCC 23726, a genetically tractable and biomedically important strain and, in addition, reveal that the previous F. nucleatum subsp. nucleatum ATCC 25586 genome assembly contains a 452-kb genomic inversion that has been corrected using our sequencing and assembly pipeline. To enable genomic analyses by the scientific community, we concurrently used these genomes to launch FusoPortal, a repository of interactive and downloadable genomic data, genome maps, gene annotations, and protein functional analyses and classifications. In summary, this report provides detailed methods for accurately sequencing, assembling, and annotating *Fusobacterium* genomes, while focusing on using open-source software to foster the availability of reproducible and open data. This resource will enhance efforts to properly identify virulence proteins that may contribute to a repertoire of diseases that includes periodontitis, preterm birth, and colorectal cancer.

**IMPORTANCE** *Fusobacterium* spp. are Gram-negative, oral bacteria that are increasingly associated with human pathologies as diverse as periodontitis, preterm birth, and colorectal cancer. While a recent surge in F. nucleatum research has increased our understanding of this human pathogen, a lack of complete genomes has hindered the identification and characterization of associated host-pathogen virulence factors. Here we report the first eight complete *Fusobacterium* genomes sequenced using an Oxford Nanopore MinION and Illumina sequencing pipeline and assembled using the open-source program Unicycler. These genomes are highly accurate, and seven of the genomes represent the first complete sequences for each strain. In summary, the FusoPortal resource provides a publicly available resource that will guide future genetic, bioinformatic, and biochemical experiments to characterize this genus of emerging human pathogens.

## Abstract (precis)

*Fusobacterium* spp. are Gram-negative, oral bacteria that are increasingly associated with human pathologies as diverse as periodontitis, preterm birth, and colorectal cancer. While a recent surge in F. nucleatum research has increased our understanding of this human pathogen, a lack of complete genomes has hindered the identification and characterization of associated host-pathogen virulence factors. Here we report the first eight complete *Fusobacterium* genomes sequenced using an Oxford Nanopore MinION and Illumina sequencing pipeline and assembled using the open-source program Unicycler. These genomes are highly accurate, and seven of the genomes represent the first complete sequences for each strain. In summary, the FusoPortal resource provides a publicly available resource that will guide future genetic, bioinformatic, and biochemical experiments to characterize this genus of emerging human pathogens.

## INTRODUCTION

Multiple *Fusobacterium* species are oral pathogens that can infect a broad range of human organ and tissue niches (1, 2). Fusobacterium nucleatum has recently been connected with colorectal cancer (CRC) (3, 4), with studies showing that this bacterium induces a proinflammatory tumor microenvironment (5, 6) and chemoresistance against drugs used to treat CRC (7). Despite the importance of this bacterium in human diseases, there is a lack of complete genomes from biomedically relevant isolates for virulence factor identification. Further motivation for complete sequencing and assembly of a library of *Fusobacterium* genomes came from the observation that our bioinformatic analyses frequently uncovered a high percentage of large predicted secreted proteins (~3,000 to 11,000 bp) encoded in the F. nucleatum subsp. nucleatum ATCC 23726 genome that were missing critical protein domains at either the N or C terminus (e.g., N-terminal Sec signal sequences).

The genome of F. nucleatum subsp. nucleatum ATCC 25586, which is the standard F. nucleatum reference strain, was completed in 2002 using cosmid and λ phage technologies to achieve long (10-to-35-kb) insertions into cosmids and to facilitate genome assembly (8). More recently, several bacterial draft genomes have been sequenced using short-read technologies (Illumina, 454 Life Sciences), and yet many of these genomes, including those from *Fusobacterium*, are incomplete multicontig builds, presumably because using only short reads makes complete genome assembly difficult due to the presence of repeat regions (e.g., CRISPR arrays, transposons). Because of this, Illumina sequencing alone is not optimal for *de novo* assembly of whole genomes because of read length limitations (~150 bp) and is better suited for guided assembly and read mapping when paired with longer-read technologies. With the emergence of next-generation long-read sequencing (Pacific Biosciences, Oxford Nanopore Technologies MinION), assembling whole genomes is now becoming standard and affordable in academic research settings. The recent combination of MinION long-read and Illumina short-read technologies to scaffold and polish DNA sequencing (DNA-seq) data, respectively, has created a robust pipeline for microbial genome completion and subsequent gene identification and characterization (9, 10). A follow-up publication by those scientists detailed their methods for concurrently sequencing 12 *Klebsiella* genomes through multiplex sampling. Following this experimental road map, we outline our detailed methods for the first completely sequenced, assembled, and annotated *Fusobacterium* genomes using MinION technology. In addition, these inaugural genomes were used to launch the FusoPortal genome and bioinformatic analysis repository (http://fusoportal.org). In summary, this report provides key resources to further determine how multiple *Fusobacterium* species contribute to a variety of human infections and diseases.

## RESULTS AND DISCUSSION

### Genome sequencing and assembly.

**FIG 1:**
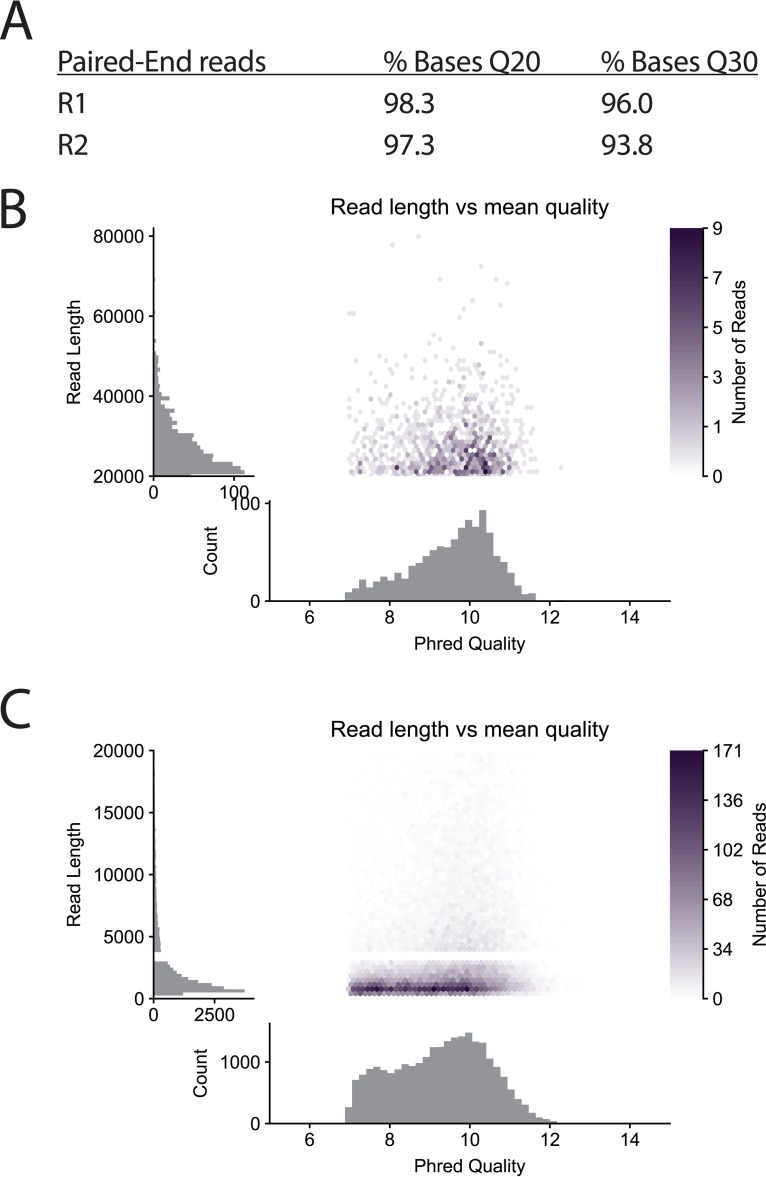
Phred analysis of Illumina and MinION reads for the F. nucleatum subsp. nucleatum ATCC 25586 genome. (A) *Q* scores for Illumina reads. (B) *Q* scores (Phred quality) for MinION reads 20 to 80 kb in length. (C) *Q* scores for MinION reads up to 20 kb in length.

Here we present eight complete *Fusobacterium* genomes; seven are the first complete genomes for each strain, and one, the genome of F. nucleatum subsp. nucleatum ATCC 25586, is a new, complete assembly that corrects a previously missed 452-kb inversion. Completion of each genome was achieved with standard DNA purification techniques, and sequencing and assembly were completed without the need for supercomputing resources. We note that these genomes range in size from 1.6 to 3.5 Mb and that larger bacterial genomes with greater numbers of repeats could require different computing needs. By sequencing multiple barcoded strains at the same time in both Illumina and MinION sequencing runs, costs were reduced to below $250 per completed genome. We show the raw sequencing data to be of high quality (Fig. 1), with high Phred scores (*Q* scores) for both Illumina and MinION reads. We report additional sequencing statistics in Tables S1, S2, and S3 in the supplemental material and highlight that the mean depth of coverage was 53× to 336× (Illumina) or 12× to 70× (MinION).

10.1128/mSphere.00269-18.1 TABLE S1  Statistics for short-read Illumina sequencing. Download TABLE S1, PDF file, 0.1 MB. Copyright © 2018 Todd et al. 2018 Todd et al. This content is distributed under the terms of the Creative Commons Attribution 4.0 International license.

10.1128/mSphere.00269-18.2 TABLE S2  Experimental details of MinION sequencing. Download TABLE S2, PDF file, 0.1 MB. Copyright © 2018 Todd et al. 2018 Todd et al. This content is distributed under the terms of the Creative Commons Attribution 4.0 International license.

10.1128/mSphere.00269-18.3 TABLE S3  Results of MinION sequencing. Download TABLE S3, PDF file, 0.1 MB. Copyright © 2018 Todd et al. 2018 Todd et al. This content is distributed under the terms of the Creative Commons Attribution 4.0 International license.

**FIG 2:**
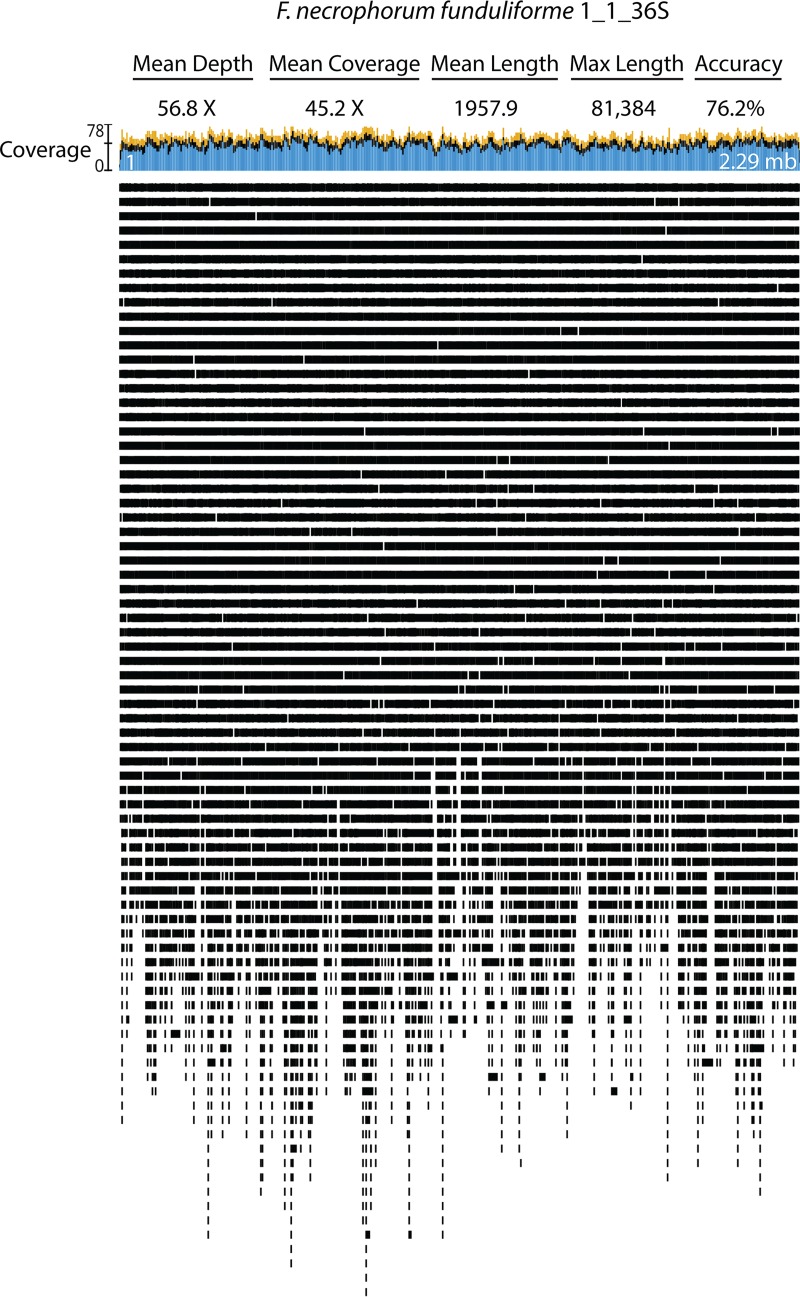
Statistics and mapping of F. necrophorum *funduliforme* 1_1_36S MinION long reads. After complete genome assembly, MinION reads were mapped to the F. necrophorum *funduliforme* 1_1_36S genome using Geneious version 9.1.4 software.

Those ranges of depth of coverage proved to be sufficient and robust using the Unicycler genome assembly software package (10). Of the eight *Fusobacterium* genomes sequenced, only F. varium 27725 included a newly identified 42-kb plasmid that contains 70 protein-encoding open reading frames. In an attempt to create consistency in genomic builds, we rotated the start site of each chromosome to represent the MreC gene (gene FN1496) as it was originally the first gene of the F. nucleatum subsp. nucleatum ATCC 25586 genome build (NCBI GCA_000007325.1). Figure 2 highlights how long reads produced by MinION sequences are able to scaffold and effectively cover a bacterial genome. For the 2.29-Mb genome of F. necrophorum *funduliforme* 1_1_36S, the maximum read length was over 81 kb, with the mean depth of coverage at 56.8× (Fig. 2; see also Table S3).

### Open reading frame predictions from complete *Fusobacterium* genomes.

**FIG 3:**
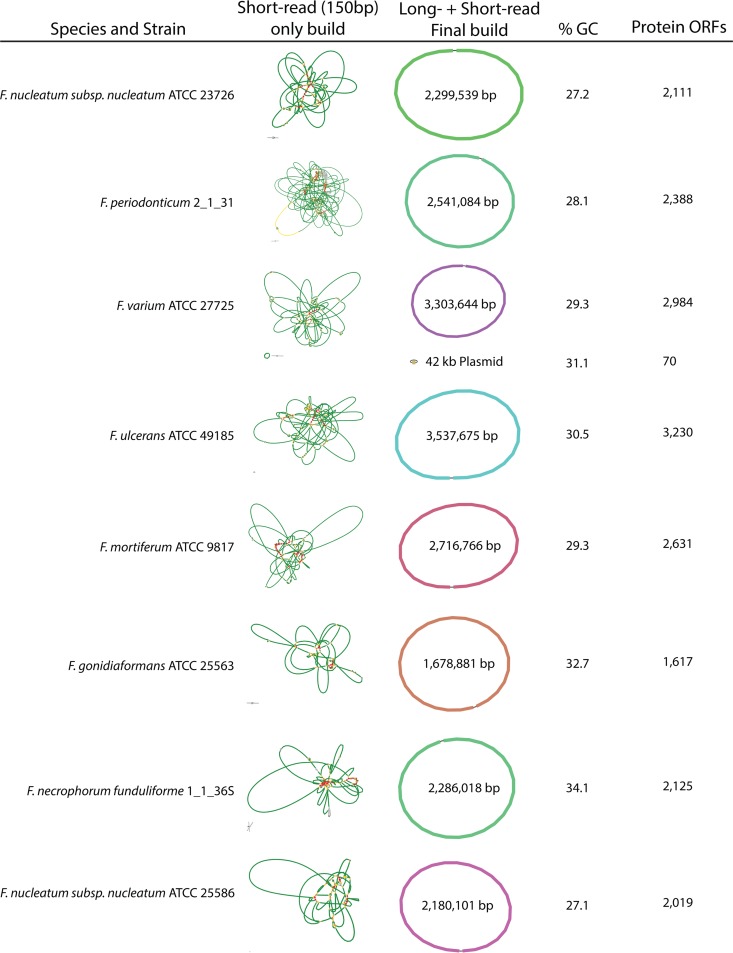
Genome assembly and annotation of eight *Fusobacterium* genomes from seven species. Short-read-only and complete genome assembly representations were created using Bandage (21).

Figure 3 depicts how using both Illumina and MinION data to assemble *Fusobacterium* genomes results in single-chromosome, complete genomes, whereas a comparative build in Unicycler using only short-read Illumina data produces multiple contig assemblies (as seen in the left column in Fig. 3). We predicted open reading frames for both protein and RNA and additionally determined all CRISPR elements for each genome. These data can found in easily searchable and downloadable formats on the FusoPortal website under their respective genomes. We highlight in detail in a companion paper that these genomes are much more accurate for annotating large genes (>3 kb), many of which belong to the type 5 secreted autotransporter family of validated virulence proteins (11, 12, 13).

### Alignment of previous draft contigs from F. nucleatum subsp. nucleatum ATCC 23726 with the complete genome.

**FIG 4:**
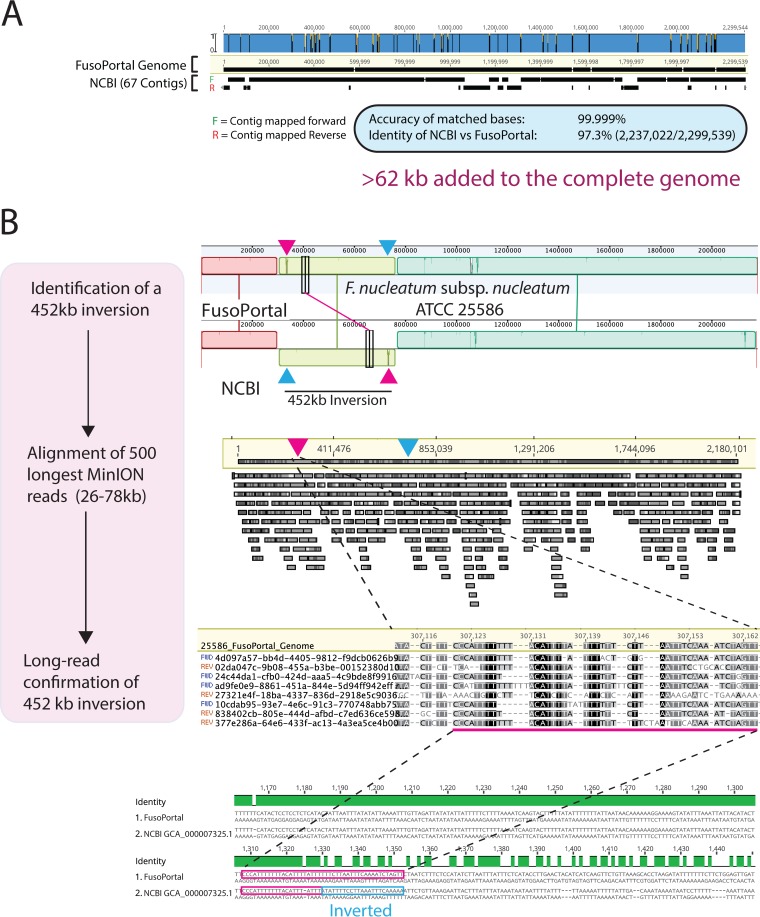
Analysis of Fusobacterium nucleatum ATCC 23726 genomes compared to previous builds. (A) Alignment of the complete F. nucleatum subsp. nucleatum ATCC 23726 genome with the 67-contig draft assembly (GenBank accession number ADVK01000000). (B) Confirmation of a 452-kb genomic inversion in the previous F. nucleatum subsp. nucleatum ATCC 25586 genome assembly (GenBank accession number GCA_000007325.1).

To highlight the effectiveness of our genome assembly pipeline, Fig. 4A shows the alignment of 67 contigs from the previous F. nucleatum subsp. nucleatum ATCC 23726 draft genome with our completed circular genome. We show that all of the contigs mapped, with our genome completing previous gaps. The new build adds 62 kb to the completed genome, and we show in a companion manuscript that this results in the correction of a significant portion of previously misannotated genes around contig ends (13). The accuracy of our genome compared to mapped base pairs from the draft genome assembly at NCBI (GCF_000178895.1) shows 99.99% base identification as determined by Geneious version 9.1.4.

### Correction of the F. nucleatum subsp. nucleatum ATCC 25586 genome.

To test the accuracy of our genomics pipeline, we chose to sequence the well-characterized strain F. nucleatum subsp. nucleatum ATCC 25586, whose sequencing was previously completed and reported in the year 2002 (8). On the basis of the results of Geneious alignment using the Mauve plugin, (14), we report that our F. nucleatum subsp. nucleatum ATCC 25586 genome corrects a previously missed 452-kb genomic inversion (Fig. 4B) in the previously completed genome deposited at NCBI (GCA_000007325.1). This region is flanked on both ends by ~8-kb repeats that are likely the reason for the previous inability to discover this genomic feature. To validate this inversion, we aligned eight MinION reads (30 to 68 kb) that spanned this region and showed that those sequences confirm this genomic correction.

### Conclusion.

The rapid evolution of DNA sequencing technologies has driven prices and computational power requirements lower at an impressive rate. The development of cost-efficient long- and short-read sequencing, in combination with open-source software for genome assembly and annotation, is igniting a revolution in bacterial genomics. We have used a previously validated pipeline for sequencing and annotation and applied this to create a library of *Fusobacterium* genomes. Drafts of these genomes previously consisted of 6 to 67 contigs, and in many cases we found that these draft genomes contained errors in open-reading frame annotations of long genes (>3 kb) (13). The newly completed genomes presented in this report are highly accurate, consist of one complete chromosome, and are freely available on our newly initiated FusoPortal website. In the future, we will use this pipeline with increased sequence multiplexing, with the goals of further reducing genome sequencing costs and adding to the number of *Fusobacterium* genomes available in the FusoPortal website. Our goal is to continually expand this technology and genomic database to provide the community with accurate genomes to identify previously missed virulence proteins in the *Fusobacterium* genus of emerging opportunistic pathogens.

## MATERIALS AND METHODS

The methods described here are expanded versions of those found in our related work (13), which describes the FusoPortal genome repository.

### Bacterial growth and genomic DNA preparation.

All strains of *Fusobacterium* were grown overnight in CBHK (Columbia broth, hemin [5 µg/ml], menadione [0.5 µg/ml]) at 37°C in an anaerobic chamber (90% N_2_, 5% CO_2_, 5% H_2_). Genomic DNA from stationary-phase bacteria was isolated in deionized water (diH_2_O) from each strain using a Wizard isolation kit (Promega) and was quantitated using a Qubit fluorimeter (Life Technologies, Inc.).

### Short-read Illumina sequencing.

Short-read DNA sequencing was carried out at the Genomic Sequence Center at the Virginia Tech Biocomplexity Institute and Novogene (strain F. nucleatum subsp. nucleatum ATCC 25586). For sequencing at Virginia Tech, DNA sequencing (DNA-seq) libraries were constructed using a PrepX ILM 32i DNA library reagent kit on an Apollo 324 NGS library preparation system. Briefly, 150 ng of genomic DNA was fragmented to 400 bp using a Covaris M220 focused ultrasonicator. The ends were repaired, and an “A” base was added to the 3′ end for ligation to the adapters, which have a single “T” base overhang at their 3′ end. Following ligation, the libraries were amplified by 7 cycles of PCR and barcoded. The library generated was validated by the use of an Agilent TapeStation and quantitated using a Quant-iT dsDNA HS kit (Invitrogen) and quantitative PCR (qPCR). The libraries were then pooled and sequenced using a NextSeq 500/550 Mid Output kit V2 (300 cycles) (P/N FC-404-2003) to 2 × 150 cycles. BCL files were generated using Illumina NextSeq control software v2.1.0.32 with real-time Analysis RTA v2.4.11.0. BCL files were converted to FASTQ files, and adapters were trimmed and demultiplexed using bcl2fastq Conversion Software v2.20. Illumina sequencing statistics and genome coverage are detailed in Table S1 in the supplemental material, and the public availability of the data at NCBI is detailed in Table 1.

**TABLE 1:** Data deposited at NCBI for all sequenced *Fusobacterium* strains

Species	Strain	GenBank genome accession no.	BioProject accession no.	BioSample accession no.	SRA^a^ Illumina accession no.	SRA MinION accession no.
F. nucleatum	23726	GCA_003019785.1	PRJNA433545	SAMN08501025	SRX3740879	SRX3740878
F. nucleatum	25586	GCA_003019295.1	PRJNA433545	SAMN08706662	SRX3786193	SRX3786192
F. varium	27725	GCA_003019655.1	PRJNA433545	SAMN08501142	SRX3740889	SRX3740888
F. ulcerans	49185	GCA_003019675.1	PRJNA433545	SAMN08501141	SRX3740885	SRX3740884
F. mortiferum	9817	GCA_003019315.1	PRJNA433545	SAMN08501148	SRX3740887	SRX3740886
F. gonidiaformans	25563	GCA_003019695.1	PRJNA433545	SAMN08501140	SRX3740881	SRX3740880
F. periodonticum	2_1_31	GCA_003019755.1	PRJNA433545	SAMN08501101	SRX3740877	SRX3740876
F. necrophorum	1_1_36S	GCA_003019715.1	PRJNA433545	SAMN08501105	SRX3740883	SRX3740882

a SRA, sequence read archive at NCBI.

### Long-read MinION sequencing.

Purified *Fusobacterium* genomic DNA was sequenced on a MinION sequencing device (Oxford Nanopore Technologies) using one-dimensional (1D) genomic DNA sequencing kit SQK-LSK108 according to Oxford Nanopore Technologies instructions. Multiplexed samples were barcoded using a 1D native barcoding kit (EXP-NBD103) according to instructions. Briefly, purified genomic DNA was repaired with NEBNext FFPE repair mix (New England Biolabs). A NEBNext Ultra II End-Repair/dA-tailing module was utilized to phosphorylate 5′ ends and add dAMP to the 3′ ends of the repaired DNA. For multiplexed samples, barcodes were ligated to the end-prepped DNA using NEB Blunt/TA master mix (New England Biolabs). Barcoded samples were pooled into a single reaction mixture, and an adapter (Oxford Nanopore Technologies) was ligated to the DNA using NEBNext Quick T4 DNA ligase (New England Biolabs). For single reactions, an adapter (Oxford Nanopore Technologies) was ligated to the end-prepped DNA using NEB Blunt/TA master mix (New England Biolabs). The DNA was purified with AMPureXP beads (Beckman Coulter, Inc., Danvers, MA) following each enzymatic reaction. Purified, adapted DNA was sequenced on an MK1B (MIN-101B) MinION platform with a FLO-min 106 (SpotON) R9.4 or FLO-min 107 (SpotON) 9.5 flow cell using MinKNOW software version 1.7.10 or 1.7.14 (Oxford Nanopore Technologies). After sequencing, Fast5 files were base-called using Albacore version 2.1.7 (Oxford Nanopore) on a Macbook Pro with a 3.3 GHz Intel Core i7 processor. For multiplexed samples, base-called fastq files were demultiplexed based on the ligated barcode using Porechop (https://github.com/rrwick/Porechop) and adaptors were trimmed. Sample preparation and sequencing details are presented in Table S2, and MinION sequencing statistics and genome coverage are detailed in Table S3. As an example of data quality, Fig. 2 shows the long-read coverage obtained using MinION sequences for the F. necrophorum *funduliforme* 1_1_36S genome.

### Genome assembly.

Genome assemblies were carried out using Unicycler version 0.4.3 open-source software (10), resulting in complete, single chromosomes for each of the eight sequenced genomes. While both the Illumina and MinION sequencing runs produced far more data than necessary, data sets were split to utilize ample and yet reasonable mean depth of coverage for 1.6-Mb to 3.5-Mb genomes. Prior to assembly, data were not sorted based on base call quality as judged by Phred scoring, as we show in Fig. 1 that the data are of high quality. Using the mean depth of coverage for each genome described in Tables S1 and S2, each genome can be constructed in 2 to 3 h using a standard Macbook Pro laptop (2.8 GHz Intel Core i7). The utility of Unicycler therefore signifies that it is a robust method for researchers without the need for a supercomputer to handle data processing. The details of all final assemblies are shown in Fig. 3, and the public availability of the data at NCBI is detailed in Table 1. For consistent starts to the circular chromosome, each genome was rotated to have gene 1, which encodes the rod-shape-determining protein MreC, in the reverse orientation as seen for the beginning of the F. nucleatum subsp. nucleatum ATCC 25586 reference genome (8).

### Open reading frame predictions.

Gene predictions for protein-encoding open reading frames were carried out using the bacterium-specific program Prodigal version 2.6.3 via command line on a Mac (15). Genes for tRNA encoding were predicted with Prokka (16) using the KBase server (17). rRNAs were identified using Barrnap (bacterial rRNA predictor) version 0.8 (18). In addition, we used the CRISPRone Web server (19) to identify all CRISPR-associated proteins and arrays, which consist of spacer and repeat regions. Details of each of these components are found on the FusoPortal repository. For each genome, the protein-encoding gene predictions by Prodigal and Prokka were in nearly complete agreement (data not reported). In addition, genome annotation for each genome was performed by NCBI upon data deposition into GenBank (Table 1).

### Software and code availability.

All software and scripts used in this study have been described and properly referenced in previous Materials and Methods sections.

### Technical validation of sequencing reads and whole genomes.

Phred quality scores for Illumina sequencing reads were determined using Geneious version 9.1.4, and these data are shown for the F. nucleatum subsp. nucleatum ATCC 25586 genome in Fig. 1A. In addition, MinION read quality was assessed using the software package Pauvre as depicted in Fig. 1B and C. Data for all eight genomes as seen in Fig. 1A can be found on the Fusoportal website (http://fusoportal.org/phred.html).

CheckM (20) on the Kbase server was used to check the quality of each genome using the reduced tree data set setting. Analysis for all genomes can be found on the Fusoportal website (http://fusoportal.org/checkm.html).

### Accession number(s).

Raw data and completed genomes for each of the eight *Fusobacterium* strains have been deposited at NCBI under the BioProject, BioSamples, sequence read archives (SRA), and GenBank accession numbers detailed in Table 1.

### Data availability.

The raw data, genome assemblies, and annotations can be accessed via the NCBI BioProject under accession PRJNA433545, and further details of these files can be found in Table 1. In addition, all of these data are easily accessible in our newly implemented FusoPortal data repository or on our Open Science Framework database (http://osf.io/2c8pv).
