# Supplementary material for: Fusobacterium Genomics Using MinION and Illumina Sequencing Enables Genome Completion and Correction
Source: mSphere. 2018 Jul 5;3(4):e00269-18. doi: 10.1128/mSphere.00269-18 (PMC6034080; doi:10.1128/mSphere.00269-18)
Supplement: TABLE S1 [file sph004182585st1.pdf]

Species	Strain	# Illumina Sequences	Base Pairs	Mean Length	Max Length	Genome Size	Mean Depth
F. nucleatum	23726	5,163,442	774.5 mb	150.2	151	2,299,539	336 X
F. nucleatum	25586	1,250,000	187.5 mb	150.0	150	2,180,101	86.0 X
F. varium	27725	1,250,000	187.5 mb	150.2	151	3,303,644 Plasmid: 42,814	56.8 X
F. ulcerans	49185	1,250,000	187.5 mb	150.2	151	3,537,675	53.1 X
F. mortiferum	9817	1,250,000	187.5 mb	150.2	151	2,716,766	69.2 X
F. gonidiaformans	25563	1,250,000	187.5 mb	150.2	151	1,678,881	111 X
F. periodonticum	2_1_31	1,250,000	187.5 mb	150.2	151	2,541,084	73.8 X
F. necrophorum	1_1_36S	1,250,000	187.5 mb	150.2	151	2,286,018	82.2 X
