# Supplementary material for: Fusobacterium Genomics Using MinION and Illumina Sequencing Enables Genome Completion and Correction
Source: mSphere. 2018 Jul 5;3(4):e00269-18. doi: 10.1128/mSphere.00269-18 (PMC6034080; doi:10.1128/mSphere.00269-18)
Supplement: TABLE S2 [file sph004182585st2.pdf]

Sample IDs	Sequencing Kit	Flow Cell	MinKNOW Version	Input DNA (μg)
23726	SQK-LSK108	FLO-MIN106 (SpotON) R9.4	1.7.10	2.38
25586	SQK-LSK108	FLO-MIN107 (SpotON) R9.5	1.7.14	3.96
49185, 9817, 25563, 2_1_31, 1_1_36S, 27725[#]	SQK-LSK108, EXP-NBD103	FLO-MIN107 (SpotON) R9.5	1.7.14	3.72, 3.12, 2.94, 0.6, 2.31, 1.83

[#]Multi-plexed on one flow cell.s
