# Supplementary material for: Fusobacterium Genomics Using MinION and Illumina Sequencing Enables Genome Completion and Correction
Source: mSphere. 2018 Jul 5;3(4):e00269-18. doi: 10.1128/mSphere.00269-18 (PMC6034080; doi:10.1128/mSphere.00269-18)
Supplement: TABLE S3 [file sph004182585st3.pdf]

Species	Strain	# MinION Sequences	Base Pairs	Mean Length	Max Length	Genome Size	Mean Depth
F. nucleatum	23726	13,904	81,303,330	5,847.5	55,886	2,299,539	35.4 X
F. nucleatum	25586	25,240	111,179,924	4,404.9	84,482	2,180,101	60.0 X
F. varium	27725	31,066	89,376,121	2,877.0	62,500	3,303,644 Plasmid: 42,814	27.0 X
F. ulcerans	49185	20,313	42,439,006	2,089.3	74,841	3.537,675	12.0 X
F. mortiferum	9817	37,658	118,090,402	3,135.9	83,201	2,716,766	43.5 X
F. gonidiaformans	25563	58,596	105,220,409	1,784.7	87,683	1,678,881	62.7 X
F. periodonticum	2_1_31	52,316	178,049,514	3,403.3	68,386	2,541,084	70.0 X
F. necrophorum	1_1_36S	66,335	129,875,928	1957.9	81,384	2,286,018	56.8 X
